# Ischemia/reperfusion‐induced alterations of enzymatic and signaling functions of the rat cardiac Na^+^/K^+^‐ATPase: protection by ouabain preconditioning

**DOI:** 10.14814/phy2.12991

**Published:** 2016-10-04

**Authors:** Aude Belliard, Gaurav K. Gulati, Qiming Duan, Rosana Alves, Shannon Brewer, Namrata Madan, Yoann Sottejeau, Xiaoliang Wang, Jennifer Kalisz, Sandrine V. Pierre

**Affiliations:** ^1^ Department of Biochemistry and Cancer BiologyCollege of MedicineUniversity of ToledoToledoOhio; ^2^ Marshall Institute for Interdisciplinary ResearchHuntingtonWest Virginia; ^3^ Present address: Materials Science and Engineering DepartmentUniversity of WashingtonSeattleWest Virginia; ^4^ Present address: Gladstone Institute of Cardiovascular DiseaseSan FranciscoCalifornia; ^5^ Present address: Université Lille 2Inserm U1011LilleFrance

**Keywords:** Na^+^/K^+^‐ATPase *α*1 and *α*2 isoforms, activity, ERK, Akt

## Abstract

Cardiac glycosides (CG) are traditionally known as positive cardiac inotropes that inhibit Na^+^/K^+^‐ATPase‐dependent ion transport. CG also trigger‐specific signaling pathways through the cardiac Na^+^/K^+^‐ATPase, with beneficial effects in ischemia/reperfusion (I/R) injury (e.g., ouabain preconditioning, known as OPC) and hypertrophy. Our current understanding of hypersensitivity to CG and subsequent toxicity in the ischemic heart is mostly based on specific I/R‐induced alterations of the Na^+^/K^+^‐ATPase enzymatic function and has remained incomplete. The primary goal of this study was to investigate and compare the impact of I/R on Na^+^/K^+^‐ATPase enzymatic and signaling functions. Second, we assessed the impact of OPC on both functions. Langendorff‐perfused rat hearts were exposed to 30 min of ischemia and 30 min of reperfusion. At the inotropic concentration of 50 μmol/L, ouabain increased ERK and Akt phosphorylation in control hearts. In I/R hearts, this concentration did not induced positive inotropy and failed to induce Akt or ERK phosphorylation. The inotropic response to dobutamine as well as insulin signaling persisted, suggesting specific alterations of Na^+^/K^+^‐ATPase. Indeed, Na^+^/K^+^‐ATPase protein expression was intact, but the enzyme activity was decreased by 60% and the enzymatic function of the isoform with high affinity for ouabain was abolished following I/R. Strikingly, OPC prevented all I/R‐induced alterations of the receptor. Further studies are needed to reveal the respective roles of I/R‐induced modulations of Na^+^/K^+^‐ATPase enzymatic and signaling functions in cardiomyocyte death.

## Introduction

Cardiac glycosides (CG) such as ouabain or digoxin are specific inhibitors of Na^+^/K^+^‐ATPase activity, which results in subsequent increase in intracellular Na^+^, increased Na^+^/Ca^2+^ exchange, and cardiac‐positive inotropy (Lee and Klaus 1971; Schwartz et al. 1988). Ouabain binding to the *α*‐subunit of Na^+^/K^+^‐ATPase also initiates specific intracellular signaling pathways, most notably PI3K/Akt and c‐Src/ERK in the cardiac tissue of various species (Mohammadi et al. 2001, 2003; Liu et al. 2007; Morgan et al. 2010; Bai et al. 2013; Duan et al. 2015). Based on experimental evidence reported in recent years, the concept that these intracellular events occur at low (subinotropic) concentration and lead to additional cardiac actions of CG such as hypertrophic growth, prevention of cardiac hypertrophy and failure, and protection against ischemia‐reperfusion injury has emerged (Liu et al. 2007; Pasdois et al. 2007; Pierre et al. 2007; D'Urso et al. 2008; Morgan et al. 2010; Bai et al. 2013; Belliard et al. 2013; Duan et al. 2015; Wu et al. 2015). The most documented example of protection against ischemia‐reperfusion injury by a CG is ouabain preconditioning (OPC). OPC can be triggered by a transient exposure to a low concentration of ouabain prior to a sustained ischemia, and provides a protection against infarction and contractile defects comparable to that observed for ischemic preconditioning. Known signaling events downstream from Na^+^/K^+^‐ATPase that are critical to OPC include the activation of Src kinase and PKC*ε*, opening of mitoK‐ATP channels, production of ROS, and activation of PI3K‐IA.

The alteration of cardiac Na^+^/K^+^‐ATPase enzyme function during ischemia and reperfusion injury is well documented (Beller et al. 1976; Kim et al. 1983; Maixent and Lelievre 1987; Lynch et al. 1988; Schwinger et al. 1999; Kjeldsen and Bundgaard 2003; Belliard et al. 2013) and is detected before cell death occurs (Inserte et al. 2005). In a model of simulated I/R in cultures of rat neonatal cardiac myocytes, we found that OPC protected Na^+^/K^+^‐ATPase catalytic properties (Belliard et al. 2013) and prevented I/R‐induced cell death. In this cell model, intrinsic Na^+^/K^+^‐ATPase enzymatic activity was protected by OPC, but the cellular uptake of the K^+^ congener ^86^Rb^+^ remained reduced. As potential explanations for this observation, we proposed the persistence of ouabain binding to the high‐affinity *α*3 isozyme expressed in rat neonatal cardiac myocytes and/or the inhibition of catalytically competent Na^+^/K^+^‐ATPase by the labile cytosolic compound described by Fuller et al. (2003). As an important implication of this finding, the fact that protection of Na^+^/K^+^‐ATPase‐mediated ion‐transport was not critical for OPC‐mediated cell survival during I/R in this model led us to propose that alteration of Na^+^/K^+^‐ATPase signaling function during I/R and protection by OPC could be involved. As a follow up, this study was undertaken to thoroughly assess how I/R and OPC impact Na^+^/K^+^‐ATPase enzymatic and signaling functions in the whole heart. The results obtained in Langendorff rat heart preparations exposed to 30 min of global ischemia followed by 30 min of reperfusion revealed marked functional changes of *α*2‐containing Na^+^/K^+^‐ATPase isoenzymes, with concomitant blunt of both contractile and signaling responses to ouabain but not dobutamine or insulin. Preconditioning using a subinotropic concentration of ouabain prevented ischemia‐induced alterations of Na^+^/K^+^‐ATPase enzymatic and signaling properties.

## Methods

### Rat heart Langendorff preparation

Experimental procedures were conducted in accordance with the *Guide for the Care and Use of Laboratory Animals* (NIH Publication, 8th Edition, 2011). A rat heart preparation perfused at constant flow in the Langendorff mode was used as previously described (Pierre et al. 2007). Male Sprague–Dawley rats (320–350 g, strain code 001) were anesthetized by intraperitoneal injection of sodium pentobarbital (80 mg/kg). Hearts were excised and rapidly placed into ice‐cold Krebs–Henseleit (KH) buffer. Within 40 sec, hearts were perfused in the Langendorff mode with oxygenated KH buffer containing (in mmol/L) NaCl (118.0), KCl (4.0), CaCl_2_ (1.8), KH_2_PO_4_ (1.3), MgSO_4_ (1.2), Ethylene glycol bis (2‐aminoethylether)‐N, N, N′, N′‐tetraacetic acid (0.3), NaHCO3 (25), D‐glucose (11). Isovolumic left ventricular developed pressure (LVDP) as well as end diastolic pressure (EDP) were continuously monitored during the experiment through a water‐filled latex balloon inserted into the left ventricle and analyzed subsequently using Lab Chart software (ADInstruments, Colorado Springs, CO). Hearts were paced at 4.5 Hz with bipolar electrodes attached to the left ventricle using a Grass SD9 stimulator, and pacing was maintained throughout all protocols. EDP was adjusted initially at 4–8 mmHg. After 20 min of equilibration, one of the two protocols described in the next section was initiated.

### Experimental protocols

#### Protocol A

**Figure 1:**
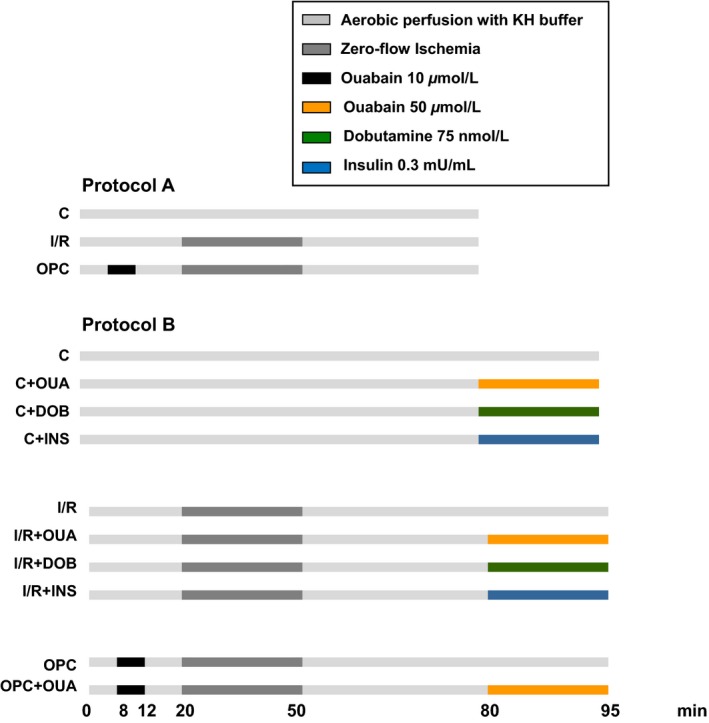
Experimental protocols. Cardiac function was recorded in control (C), ischemia‐reperfusion (I/R), and ouabain preconditioning (OPC) groups exposed to protocol A. The inotropic and signaling effects of ouabain (OUA), dobutamine (DOB), and insulin (INS) were evaluated using protocol B. KH, Krebs–Henseleit buffer.

To document the effect of ischemia and reperfusion on cardiac function, tissue viability, and Na^+^/K^+^‐ATPase structure and enzyme function, hearts were subjected to the 80 min‐long protocol A shown in Figure 1. Control hearts (C) were perfused with KH buffer for 80 min. Ischemia/reperfusion (I/R) hearts were perfused for 20 min with KH, then subjected to 30‐min zero‐flow (global) ischemia followed by 30 min of reperfusion. Ouabain preconditioning (OPC) hearts were perfused with KH for 8 min followed by ouabain (10 μmol/L) for 4 min and KH for 8 min before 30 min of global ischemia and 30 min of reperfusion as previously described (Pierre et al. 2007). At the end of 30 min of reperfusion, left ventricles were snap frozen in liquid nitrogen and stored at −80°C for Na^+^/K^+^‐ATPase activity measurement and western blot analysis of *α*_1_, *α*_2_, and *β*_1_ isoforms of Na^+^/K^+^‐ATPase.

#### Protocol B

To investigate the effect of ischemia and reperfusion on ouabain‐induced positive inotropy and signaling, a 15 min exposure to KH or 50 μmol/L ouabain was added to one of the 80 min protocols described above (i.e., C, I/R, or OPC). A subset of hearts was exposed to 15 min dobutamine (75 nmol/L) or insulin (0.3 mU/mL) after the initial 80 min period as shown in Figure 1, and used as controls in inotropy and signaling studies.

### Lactate dehydrogenase activity measurement

Coronary effluents were collected for 30 sec at time 0, 1, 2, 3, 4, 5, 10, 15, 20, and 30 min of reperfusion. Lactate dehydrogenase (LDH) activity was determined colorimetrically using a standard assay kit (Cytotoxicity detection kit (LDH), Version 8.0; Roche Diagnostics, Indianapolis, IN), according to manufacturer recommendation.

### Crude heart homogenates (KCl‐enzyme preparations)

Sample preparation was performed following a tissue homogenization protocol modified from (Huang et al. 1979). Briefly, 200 mg of left ventricular liquid‐nitrogen processed tissue powder were dissolved in 10 mL of 1 mol/L KCl solution and homogenized with a Polytron PT‐10/ST for 30 sec. This method of homogenization coupled with a KCl treatment allows the disruption of the myofilament contractile proteins present in the fraction. The homogenate was passed through a double‐layered cheese cloth and centrifuged at 1000 *g* for 10 min. The pellet was suspended in 14 mL of 50 mmol/L KCl and 50 mmol/L Tris HCl (pH 7.4) and centrifuged again. This washing procedure was repeated twice with 50 mmol/L Tris‐HCl (pH 7.4). The final pellet was suspended in 1 mL of 1 mmol/L Tris‐EDTA (pH 7.4) and the suspension was homogenized with a Potter‐Elvehjen homogenizer. All procedures were conducted on ice and using a refrigerated centrifuge at 4°C.

### Na^+^/K^+^‐ATPase activity

Na^+^/K^+^‐ATPase activity was measured by colorimetric determination of inorganic phosphate released from ATP. To insure access of substrates and inhibitor to both the ATP‐ and ouabain‐binding sites of Na^+^/K^+^‐ATPase enzymes in closed membrane vesicles that may have formed during the procedure, KCl‐enzyme preparations were pretreated with alamethicin (0.05 mg/mg of protein) for 10 min at room temperature as described previously (Xie et al. 1989). The reaction mixture for the activity assay contained 20 mmol/L Tris‐HCl (pH. 7.2), 3 mmol/L MgCl_2_, 100 mmol/L NaCl, 20 mmol/L KCl, 1 mmol/L EGTA‐Tris, 5 mmol/L NaN_3_, 2 mmol/L ATP, the KCL‐enzyme preparation, and 1 mmol/L ouabain when indicated. After addition of Mg^2+^/ATP, the enzymatic reaction was allowed to run for 10 min. The reaction was terminated by addition of 1 mL cold 8% trichloroacetic acid and rapid placement of each test tube on ice. Using an inorganic phosphate detection kit (Biomol Green; Enzo Life Sciences, Farmingdale, NY), released phosphate was quantified spectrophotometrically at 620 nm. For each sample, the assay was done in the presence or absence of 1 mmol/L ouabain to determine ouabain‐insensitive and total Na^+^/K^+^‐ATPase activity, respectively. Ouabain‐sensitive Na^+^/K^+^‐ATPase activity was then calculated as the difference between ouabain‐insensitive and total Na^+^/K^+^‐ATPase activity.

### Effect of various concentrations of ouabain on Na^+^/K^+^‐ATPase activity

Ouabain‐sensitive Na^+^/K^+^‐ATPase activity was determined in KCL‐enzyme preparation in the presence of 10^−8^ to 10^−3^ mol/L ouabain in the reaction mixture. IC_50_ (50% inhibitory constant) and relative proportion of *α*_1_ and *α*_2_ isoform of Na^+^/K^+^‐ATPase was inferred from ouabain‐sensitive Na^+^/K^+^‐ATPase activity, as estimated from dose–response curves on permeabilized crude heart homogenate preparations using GraphPad PRISM Software (Version 4.00). Curves were fit to experimental data by a nonlinear regression model using GraphPad PRISM Software (San Diego, CA).

### Tissue preparation, SDS‐PAGE, and Western blots

One hundred milligram of powdered left ventricle was added to an ice‐cold buffer containing 1 mmol/L EDTA, 1 mmol/L phenylmethylsulfonyl fluoride, 1 mmol/L sodium orthovanadate, 1 mmol/L NaF, 10 μmol/L okadaic acid, 10 μg/mL aprotinin, 10 μg/mL leupeptin and homogenized in a 30 mL homogenizer by repeating five times a series of eight up‐and‐down strokes separated by 30 sec intervals, and rotating for 90 min at 4°C. Lysates were then centrifuged at 16,000 × *g* for 15 min, and supernatants were used. Equal amount of proteins were loaded and separated by 10% SDS‐PAGE, transferred onto nitrocellulose membrane (Optitran BA‐ S 83, 0.2 μmol/L, GE Healthcare Life Sciences, Pittsburgh, PA), and immunoblotted with antibodies against Na^+^/K^+^‐ATPase *α*_1_ (*α*6F; Developmental studies hybridoma bank, University of Iowa, Iowa), *α*_2_ (HERED primary‐antibody; a gift from Dr T. A. Pressley, Texas Tech University HSC, Lubbock, TX), and *β*_1_ (05‐382, Upstate, CA) of Na^+^/K^+^‐ATPase subunits, phosphorylated and total Akt (T‐Akt: #9272, Cell Signaling Tech., Danvers, MA; PAkt: S 473, Cell Signaling Tech.), ERK_1/2_ (pERK_1/2_: Sc‐7383, Santa Cruz, Santa Cruz, CA; T‐ERK_1/2_: Sc‐94, Santa Cruz) and Src (C‐Src: Sc‐8056, Santa Cruz; pSrc: 44660G, Thermofisher Scientific, Waltham, MA). GAPDH (Sc‐20357; Santa Cruz) was probed as a loading control. Rabbit (Sc‐2004; Santa Cruz), goat (Sc‐2020; Santa Cruz), and mouse (Sc‐2005; Santa Cruz) secondary antibodies were used in this study. Protein bands were detected using chemiluminescence and enhanced chemiluminescence, and quantified using Image J software (NIH, Bethesda, DC).

### Statistical analysis

All values are expressed as mean ± SEM. Comparisons between groups were conducted using a one‐way ANOVA followed by a multiple comparison post hoc test. When two groups were compared, unpaired bilateral Student's *t* test was used. *P* < 0.05 was considered statistically significant.

## Results

### Impact of I/R and OPC protocols on contractile function and tissue injury

**Figure 2:**
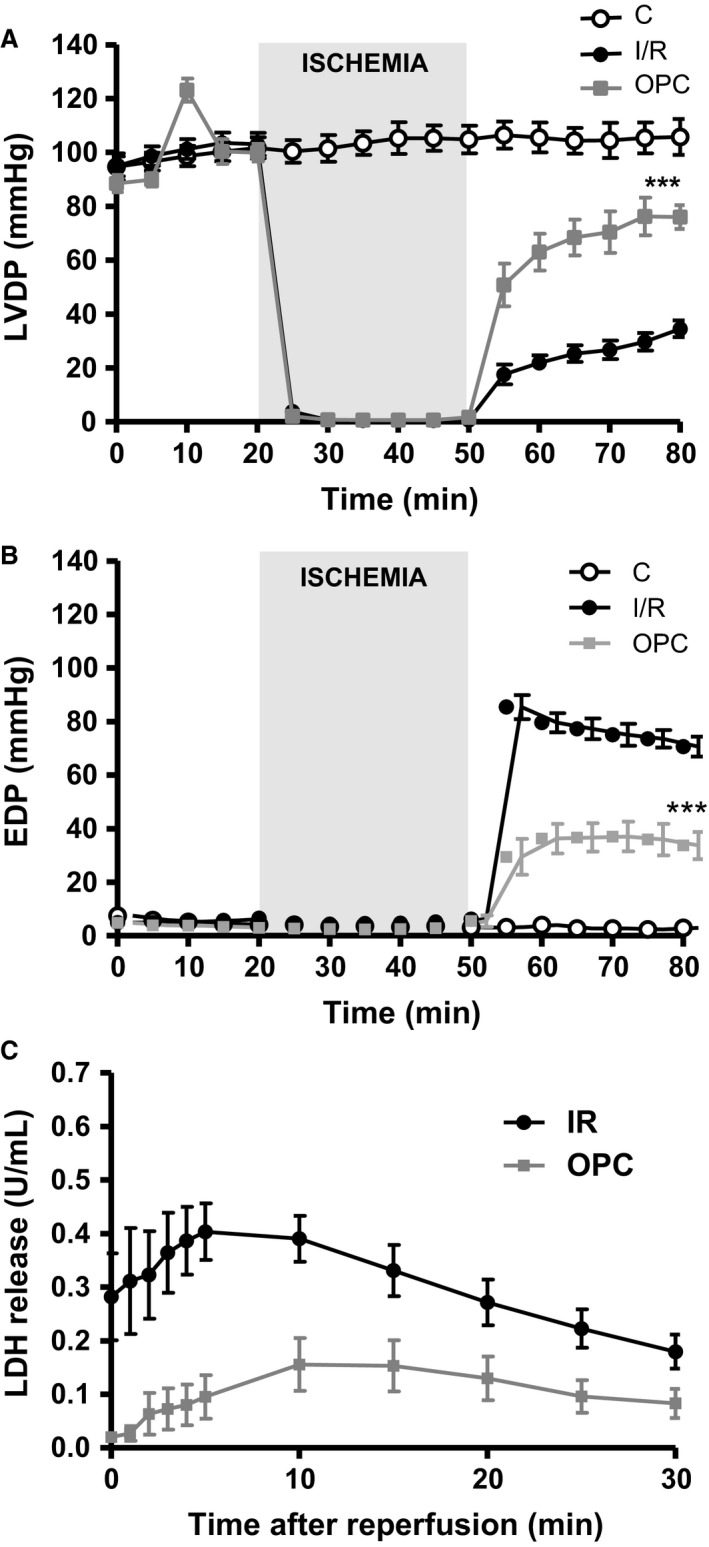
Myocardial function. Left ventricular developed pressure (LVDP, A) and end diastolic pressure (EDP, B) were continuously measured in control hearts (C), hearts exposed to 30 min of ischemia followed by 30 min of reperfusion (I/R), or hearts exposed to ouabain preconditioning (OPC) according to protocol A (Fig. 1). The amount of lactate dehydrogenase (LDH) was measured in coronary effluent during reperfusion (C). Values are means ± SEM of 6–10 independent experiments for each group.

The contractile performance of control hearts assessed by real‐time recording of LVDP and EDP was stable over 80 min of protocol A (Fig. 1), as shown in Figure 2A and B. In contrast, 30 min of ischemia followed by 30 min of reperfusion resulted in a 68% decrease in LVDP at 80 min compared to preischemic value (106 ± 7 mmHg vs. 35 ± 3 mmHg, *P *<* *0.001), and a significant increase in EDP (3 ± 2 mmHg vs. 71 ± 5 mmHg, *P *<* *0.001). OPC resulted in a significantly better recovery of LVDP (76 ± 5 mmHg vs. 35 ± 3 mmHg; *P* < 0.001) and EDP (34 ± 5 mmHg vs. 71 ± 4 mmHg; *P *<* *0.001) at 80 min compared to I/R. As shown in Figure 2C, the protective effect of OPC was also associated with a 70% reduction in LDH release (0.08 ± 0.03 U/mL vs. 0.18 ± 0.03 U/mL; *P *<* *0.05). Taken together, the above findings were consistent with our early report of OPC‐induced protection in this model (Pierre et al. 2007).

### Na^+^/K^+^ ATPase structure and enzyme function

**Figure 3:**
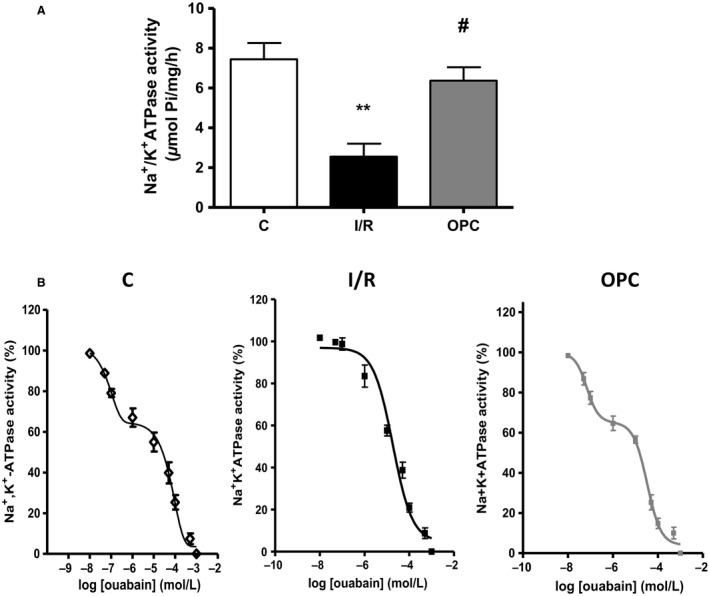
Na^+^/K^+^‐ATPase enzymatic properties in crude heart homogenates. (A) Na^+^/K^+^‐ATPase in hearts exposed to protocol A. Values are means ± SEM of eight independent experiments for each group. C, control; I/R, ischemia/reperfusion; OPC, ouabain preconditioning. **P < 0.01 versus C, ^#^ *P < *0.05 versus I/R. (B) Ouabain dose‐dependent inhibition of Na^+^/K^+^‐ATPase. Values are means ± SEM (*n* = 6). Lines represent the theoretical curves after analysis using a nonlinear regression model (see Methods) best fitted with a two sites model (control and OPC) or a one site model (I/R). Computed IC_50_ and contributions of each site are reported in Table 1.

**Figure 4:**
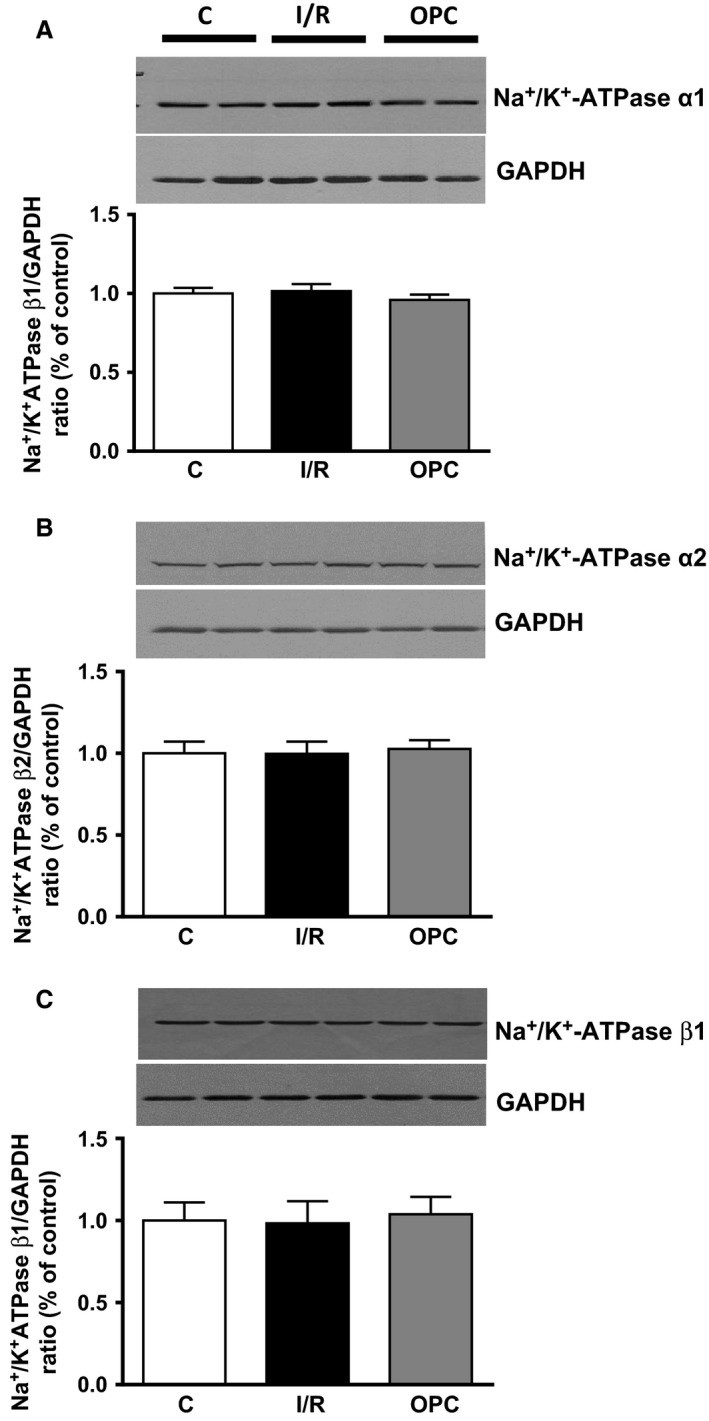
Effect of ischemia/reperfusion and OPC on Na^+^/K^+^‐ATPase subunits. Upper panels*:* representative immunoblots of Na^+^/K^+^‐ATPase *α*1 (A), *α*2 (B), and *β*1 (C). Lower panels: quantitative analysis of immunoblots. Values are means ± SEM of 8–10 independent samples for each group, expressed as subunit/GAPDH ratios. These ratios are normalized to one C sample/gel, which was assigned the value of 1. C, control; I/R, ischemia/reperfusion; OPC, ouabain preconditioning.

As shown in Figure 3A, the ouabain‐sensitive activity corresponding to Na^+^/K^+^‐ATPase in alamethicin‐treated KCl preparations from control hearts was 7.4 ± 0.8 μmol Pi/h/mg of protein. After 30 min of ischemia and 30 min of reperfusion, Na^+^/K^+^‐ATPase was decreased by 66% (2.5 ± 0.6 μmol Pi/h/mg of protein; *P *<* *0.001 vs. control) and significantly protected by OPC (*P* < 0.05 vs. I/R). Na^+^/K^+^‐ATPase in adult rat hearts typically exhibit a biphasic dose–response curve to ouabain, with a low‐affinity site attributed to *α*1‐containing isoenzymes, and a high‐affinity component attributable to *α*2‐containing isoenzymes (Maixent et al. 1999). As expected, the log concentration–effect curves were biphasic for control hearts in this study, as shown in Figure 3B (C). As represented by the solid line, dose–response curves were best modeled assuming two computed IC_50_ (ouabain concentration leading to 50% inhibition) of 5.9 × 10^−8^ mol/L and 9.1 × 10^−5^ mol/L, with respective contributions of 33% and 67% (Table 1). In contrast, dose–response curves from I/R hearts were best fitted assuming one IC_50_ rather than two, resulting in a monophasic curve (I/R). The computed IC_50_ of the remaining site was 1.8 × 10^−5^ mol/L, suggesting a loss of the high‐affinity site. OPC protected the activity of the site of high affinity, as suggested by the biphasic curve. Contributions of the two isoenzymes were 64% for the site of low affinity (*α*1) and 36% for the site of high affinity (*α*2). As shown in Figure 4, I/R‐induced alterations of Na^+^/K^+^‐ATPase activity and loss of high‐affinity enzymatic site or OPC protection were not accompanied by any detectable change in total cardiac Na^+^/K^+^‐ATPase *α*1, *α*2 or *β*1 protein contents as determined by Western blot analysis.

**Table 1:** Effect of ischemia and reperfusion on Na^+^/K^+^‐ATPase characteristics in crude heart homogenates

	Control	Ischemia/reperfusion	Ouabain preconditioning
Na^+^/K^+^‐ATPase activity (μmol Pi/h/mg of protein)	7.4 ± 0.8	2.5 ± 0.6	6.4 ± 0.7
Low‐affinity site (*α*_1_)			
IC_50_ (mol/L)	9.1 × 10^−5^	1.8 × 10^−5^	3.3 × 10^−5^
Contribution (%)	67	100	64
High‐affinity site (*α*_2_)			
IC_50_ (mol/L)	5.9 × 10^−8^	–	6.9 × 10^−8^
Contribution (%)	33	–	36

Values are means ± SEM (*n* = 6).

### Effect of ouabain 50 μmol/L on contractility and signaling

**Figure 5:**
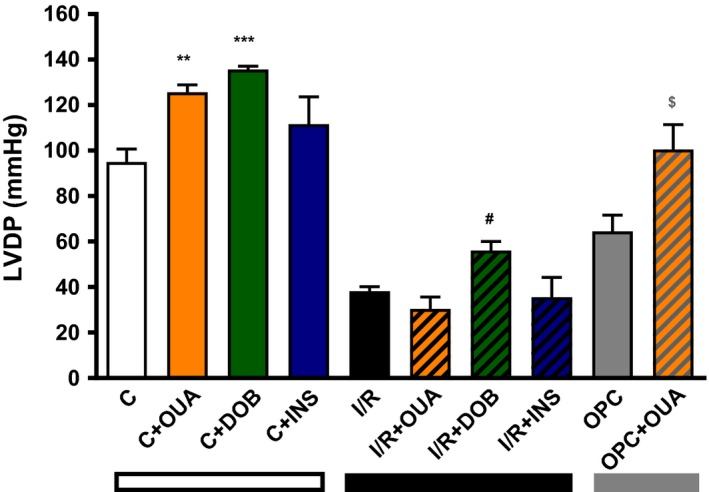
Effect of ouabain 50 μmol/L on contractile function in control, I/R and OPC hearts. Ouabain 50 μmol/L (orange bars) or dobutamine 75 nmol/L (green bars) or insulin (0.3 mU/mL) was applied for 15 min after aerobic perfusion with Krebs–Henseleit buffer or following 30 min of ischemia and 30 min reperfusion with or without OPC, according to protocol B (Fig. 1). Shown are means ± SEM from three to eight independent experiments for each group. **P <* *0.001 and ***P <* *0.0001 versus C, ^#^ *P *<* *0.05 versus I/R, and ^$^ *P *<* *0.05 versus OPC.

We next evaluated the impact of I/R on positive inotropy and signaling induced by 50 μmol/L of ouabain. This relatively high concentration was selected because it is known to trigger a robust‐positive inotropic effect without signs of toxicity, as well as a clear activation of Na^+^/K^+^‐ATPase signaling in Langendorff preparations of rat hearts (Mohammadi et al. 2003). The effects in control, I/R, and OPC hearts were compared on LVDP to monitor changes in the force of contraction, and ERK and Akt phosphorylation as markers of activation of Na^+^/K^+^‐ATPase‐mediated signaling. As expected, a 15 min exposure to ouabain 50 μmol/L according to protocol B (Fig. 1) induced a robust and significant increase in LVDP in control hearts (95 ± 6 mmHg to 126 ± 3 mmHg; *P *<* *0.05 vs. C, Fig. 5). Also expected, the I/R protocol resulted in a significant decrease in LVDP compared to control. After I/R, exposure to ouabain 50 μmol/L for 15 min failed to induce positive inotropy (Fig. 5). Exposure to 75 nmol/L of the *β*1 adrenergic agonist dobutamine, which produced a positive inotropic response comparable to ouabain 50 μmol/L in control hearts (136 ± 1 mmHg), still produced a significant increase in contractility in the I/R hearts (56 ± 4 mmHg; *P *<* *0.05 *vs*. 38 ± 2 mmHg in I/R), suggesting that the contractile apparatus of myocytes in I/R hearts was able to respond to inotropic stimuli other than ouabain.

**Figure 6:**
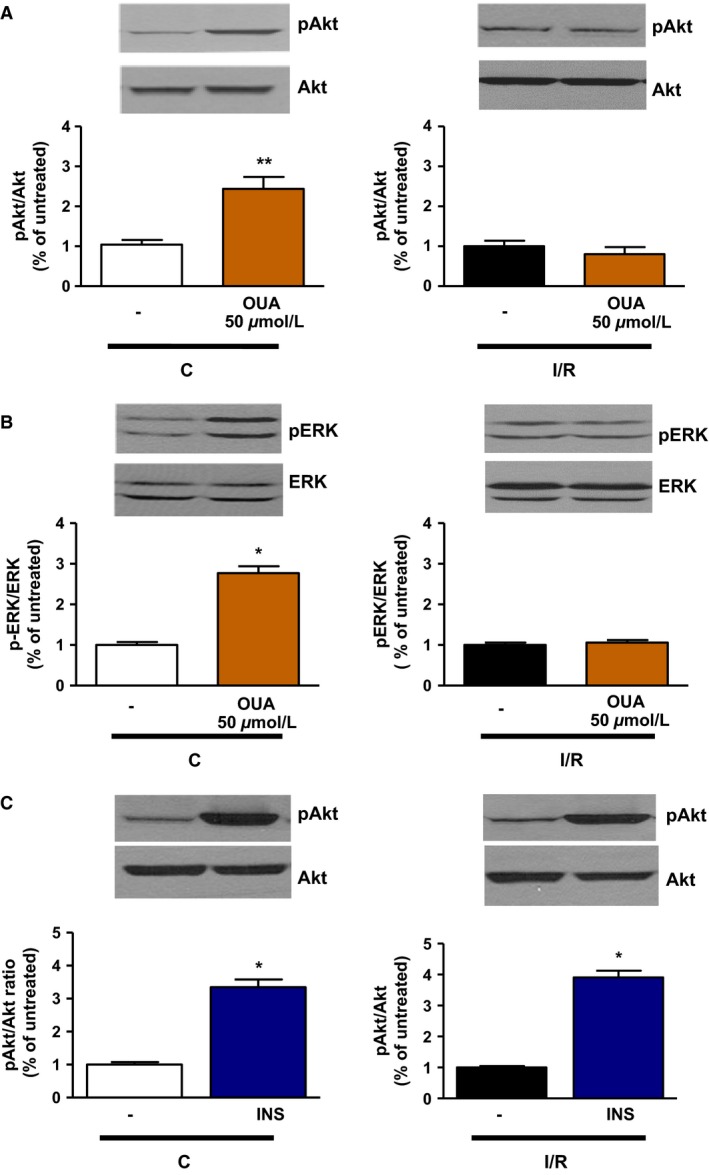
Effect of ouabain 50 μmol/L on Akt and ERK phosphorylation in control and I/R hearts. Ouabain 50 μmol/L (A, B) or insulin 0.3 mU/mL (C) was added to the perfusate for 15 min after aerobic perfusion with Krebs–Henseleit buffer or following 30 min of ischemia and 30 min reperfusion, according to protocol B (Fig. 1). Crude homogenates were assayed for total and phosphorylated forms of Akt (A) and ERK (B). Upper panels: representative immunoblots. Lower panels: activation quantified as ratio of phosphorylated to total form of the indicated protein, normalized to C (left panels) or I/R (right panels). These ratios are normalized to one C (left panels) or one I/R (left panel) sample/gel, which was assigned the value of 1. Shown are means ± SEM from three to six independent experiments. *P <* *0.05 and **P <* *0.01 versus corresponding untreated condition.

**Figure 7:**
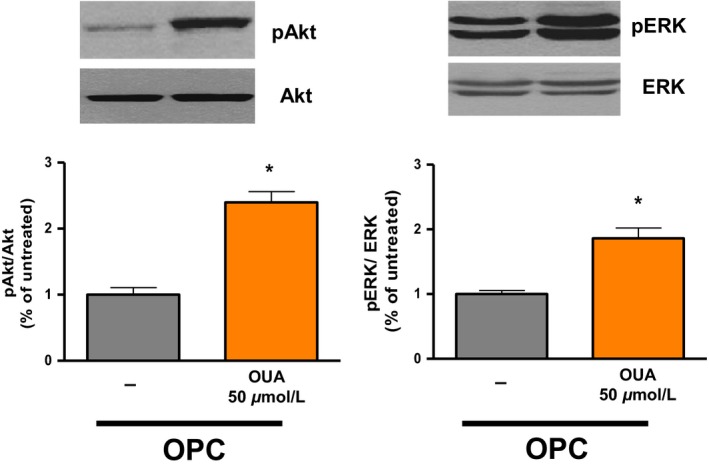
Effect of ouabain 50 μmol/L on Akt phosphorylation and ERK phosphorylation in hearts preconditioned with ouabain. Total and phosphorylated forms of Akt (left) and ERK (right). Upper panels: representative immunoblots. Lower panels: activation quantified as ratio of phosphorylated to total form of the indicated protein. These ratios are normalized to one OPC sample/gel, which was assigned the value of 1. Shown are means ± SEM from 3three to eight independent experiments. *P <* *0.05 versus OPC.

As shown in Figure 6A (left panel), ouabain 50 μmol/L resulted in a robust increase in Akt Ser‐473 phosphorylation in control hearts (2.44 ± 0.29 in OUA vs. 1.04 ± 0.11 in C; *P *<* *0.01), but not in I/R hearts (Fig. 6A, right panel). Similarly, ouabain 50 μmol/L significantly increased ERK_1/2_ phosphorylation in control hearts (1.00 ± 0.07 vs. 2.77 ± 0.17; *P *<* *0.05), but not in I/R hearts. To test whether the lack of Akt response to ouabain in I/R hearts resulted from a general defect of Akt pathway after I/R, insulin 0.3 mU/mL was used according to Protocol B (Fig. 1). As shown in Figure 5 (blue bars), this treatment did not significantly affect LVDP in control or I/R hearts, but induced a robust activation Akt Ser‐473 phosphorylation in both control and I/R hearts (Fig. 6C). OPC preserved inotropic and signaling response to ouabain 50 μmol/L. Indeed, a significant increase in LVDP was observed (64 ± 7 mmHg vs. 100 ± 11; *P* < 0.05; Fig. 5), which was accompanied by a significant increase in pAkt/Akt and pERK_1/2_/ERK_1/2_ ratios (Fig. 7).

## Discussion

In Langendorff‐perfused rat heart preparations, we examined how I/R impacts Na^+^/K^+^‐ATPase‐mediated signaling in response to 50 μmol/L of the CG ouabain. Na^+^/K^+^‐ATPase structure, signaling and enzyme function, as well as cardiac contractile function were evaluated. The results suggest that I/R induces isoform‐specific Na^+^/K^+^‐ATPase alterations, which in turn modulate ouabain inotropic and signaling response in the ischemic heart. They further revealed that OPC provides protection against all I/R‐induced alterations of Na/K‐ATPase functions.

### I/R‐induced alteration of Na^+^/K^+^‐ATPase isoenzymes and protection by OPC

Numerous studies including ours have shown decreased sodium pump activity in cardiac tissue subjected to I/R (Beller et al. 1976; Inserte et al. 2005; Singh et al. 2012; Belliard et al. 2013). In this study, Na^+^/K^+^‐ATPase in crude homogenates was reduced by over 60%, without detectable changes in total protein expression of Na^+^/K^+^‐ATPase *α*1, *α*2 or *β*1 (Figs 3A and 4). This is in keeping with results from our recent study in rat neonatal cardiac myocytes (Belliard et al. 2013), as is the complete protection provided by OPC. In the adult rat cardiac tissue, it is well established that *α*1 represents 80% of the Na^+^/K^+^‐ATPase catalytic‐subunit expressed, and has a low affinity for ouabain. Na^+^/K^+^‐ATPase *α*2 represents the remaining 20%, with a higher affinity (Lucchesi and Sweadner 1991; Gerbi et al. 1997). Consistently, ouabain dose–response curves were best fitted to a model with two phases of inhibition in control hearts, with a major contribution of the component of low affinity (Table 1). Strikingly, ouabain inhibition curves were no longer biphasic in postischemic hearts (Fig. 3B). The unique inhibitory constant was of low affinity, and the function of the high‐affinity component related to *α*2‐containing isoenzymes was no longer detected. Mechanistically, a combined exposure to high levels of reactive oxygen species (ROS) and Ca^2+^ during I/R could explain these isoenzyme‐specific alterations without change in total protein expression. Indeed, ROS preferentially alter *α*2‐mediated Na^+^/K^+^‐ATPase activity compared to *α*1 (Xie et al. 1990, 1995) a selectivity that is most likely based on structural features. It has also been known since the 1980s that Ca^2+^ levels critically affect ouabain dose–responses of cardiac Na^+^/K^+^‐ATPase. Specifically, the function of the component of high affinity is lost at supraphysiological concentrations (Mansier and Lelievre 1982; Lelievre et al. 1984). While all preparations were perfused using a physiological concentration of 1.5 mmol/L Ca^2+^ in this study, a substantial I/R‐induced increase in Ca^2+^ likely explains, at least in part, the monophasic ouabain dose–response in I/R hearts. Irrespective of the mechanism involved, this study also revealed that OPC prevented these I/R‐induced isoform‐specific alterations.

### Contractility and signaling response to CG in the I/R heart

After I/R, positive inotropy was observed in response to dobutamine but not ouabain (Fig. 5). Rather, ouabain 50 μmol/L further reduced LVDP, an adverse effect suggestive of toxicity. Indeed, although arrythmogenic effects of toxic concentrations of CG after I/R (Kim et al. 1984; Lynch et al. 1986) were not observed in our electrically paced preparations, an increase in EDP was frequently observed upon addition of ouabain in I/R hearts, suggestive of Ca^2+^ overload (not shown). Since LVDP was calculated as the difference between LVSP and EDP, this in turn contributed to a decrease in LVDP compared to I/R alone (Fig. 5). According to the model proposed by Maixent and Lelievre (1987) the loss of inotropic response and the toxic effect of 50 μmol/L ouabain after I/R are direct consequences of I/R‐induced alterations of Na^+^/K^+^‐ATPase enzymatic properties. In their model, the component of high affinity for ouabain (*α*2) is responsible for the inotropic response, whereas inhibition of the component of low affinity (*α*1) mediates the toxic effect. Consequently, the I/R‐induced loss of function of *α*2 with increased contribution of *α*1 (see Table 1) led to the observed ouabain‐specific loss of inotropic effect and toxicity (Maixent and Lelievre 1987; Maixent et al. 1987). In terms of signaling, Akt phosphorylation persisted in response to insulin (Fig. 6C), but Akt and ERK phosphorylation no longer occurred upon exposure to 50 μmol/L ouabain after I/R (Fig. 6B), suggesting that ouabain signaling was specifically altered. OPC effectively protected Akt and ERK responses to ouabain (Fig. 7).

Taken together, these findings indicate that both enzymatic and signaling functions of Na^+^/K^+^‐ATPase are specifically altered in the ischemic heart, and that OPC preserves all parameters. Two main questions remain to be addressed. First, further studies are needed to assess the role of Na^+^/K^+^‐ATPase nonenzymatic function in I/R‐induced cardiac myocyte cell death. Indeed, our previous study in rat neonatal cardiomyocytes provided indirect evidence that I/R‐induced disruption of Na^+^/K^+^‐ATPase ‐driven ion transport may not be the primary cause of I/R‐induced cell death (Belliard et al. 2013). This was based on the observation that OPC decreased I/R‐induced cardiomyocyte death without observable protection of Na^+^/K^+^‐ATPase‐mediated ion transport (assessed by ouabain‐sensitive ^86^Rb^+^ uptake), and led us to propose that an I/R‐induced effect on the nonenzymatic function of Na^+^/K^+^‐ATPase may occur. This study provides the first evidence that at least some of Na^+^/K^+^‐ATPase‐mediated signaling pathways are indeed specifically disrupted by I/R and protected by OPC, which warrants further studies of possible mechanism involved.

## Conflict of Interest

None declared.
